# The effect of Andaliman (*Zanthoxylum acanthopodium *DC.) fruit extracted with ethanol on TNF-α and TRPA-1 levels in type II diabetes-induced mice

**DOI:** 10.5455/javar.2024.k774

**Published:** 2024-06-06

**Authors:** Boyke Marthin Simbolon, OK Yulizal, Albert Manggading Hutapea, Erwin Handoko

**Affiliations:** 1 Faculty of Medicine, Universitas Prima Indonesia, Medan, Indonesia; 2 Department of Physiology, Universitas Advent Indonesi-a, Indonesia; 3 Quality Assurance and Educational Development, Murni Teguh University, Deli Serdang, Indonesia

**Keywords:** TNF-α, TRPA-1, type 2 diabetes mellitus, Andaliman fruit extract, *Zanthoxylum acanthopodium* fruit extract

## Abstract

**Objective:**

The present study investigated the effects of Andaliman fruit extract on tumor necrosis factor-alpha (TNF-α) and transient receptor potential ankyrin-1 (TRPA-1) levels in type 2 diabetes mellitus (T2DM) mouse models induced with streptozocin (STZ) and a high-fat diet (HFD).

**Materials and Methods:**

In this research, mice were allocated into six distinct groups: normal, negative control (HFD and STZ), positive control (metformin, HFD, and STZ), and three treatment groups (HFD, STZ, and Andaliman extract at varying dosages of 100, 300, and 500 mg/kg, respectively). Body weight and blood glucose levels (BGLs) were recorded at weeks 1 (baseline), 8, 12, and 16. The levels of TNF-α and TRPA-1 were measured during the 16th week.

**Results:**

Phytochemical screening of the Andaliman extract revealed the presence of flavonoids, alkaloids, tannins, saponins, and glycosides. The one-way ANOVA revealed significantly elevated BGL at week 16 in the negative control group in comparison to the other groups (*p *< 0.05). The Kruskal-Wallis test followed by Bonferroni-corrected pairwise comparisons showed that the negative control had significantly higher TNF-α levels than the Andaliman-groups (*z* = 22.11, *p < *0*.*01). TRPA-1 was significantly higher in the negative control group compared to the treatment groups (*p* < 0.05). Furthermore, Spearman’s rho analysis revealed a statistically significant positive association between BGL and both TNF-α and TRPA-1, as well as between TNF-α and TRPA.

**Conclusion:**

Andaliman extract potentially serves as a therapy for diabetic neuropathy in T2DM by lowering BGL and inhibiting the expression of TNF-α and TRPA-1.

## Introduction

Type 2 diabetes mellitus (T2DM) remains a global health challenge due to its association with serious complications that contribute to disability and mortality. Diabetic neuropathy (DN) is among the most prevalent complications of TD2M, impacting the sensory, motor, and autonomic nervous systems [1,2]. DN can significantly affect the quality of life, leading to severe depression or death [3]. Nevertheless, despite advancements in understanding T2DM and its complications, the underlying mechanisms of DN are still poorly understood.

Emerging evidence in the pathophysiology of DN has underscored the roles of proinflammatory cytokines such as tumor necrosis factor-alpha (TNF-α) and neuroimmune interactions that drive neuropathic pain [4]. In the peripheral inflammatory model of chronic pain, neuroinflammatory and neuroimmune activation occurs following damage to the peripheral nerves and nerve roots. TNF-α, in particular, is hypothesized to be a pivotal mediator of insulin resistance and metabolic dysfunction, and its elevation is believed to influence the pathogenesis of diabetic complications directly [5–7]. Reports have shown that TNF-α can impede insulin transmission and influence glucose metabolism, possibly playing a pivotal role in the onset of obesity and insulin resistance [8–10,5,5,11]. Furthermore, recent literature has reported the association between metabolic disturbances and pain perception, implicating TNF-α as a bridge linking systemic inflammation with neurogenic inflammation, potentially through activation of various signaling cascades such as nclear factor kappa-light-chain-enhancer of activated B cells and extracellular signal-regulated kinase pathways [12].

The transient receptor potential ankyrin-1 (TRPA-1) channel has also drawn attention to its role in sensory neuron activation and mediating pain signals in DN [13]. TRPA-1 is the channel primarily found on the primary afferent nerve fibers that mediate pain [14]. Studies on mouse models of T2DM have indicated that the hyperglycemia environment of T2DM prompted the increased production of reactive metabolites, such as methylglyoxal (MGO), which act as TRPA-1 agonists. MGO exacerbates pain and hypersensitivity by activating TRPA-1 and sodium voltage-gated channel 1.7 (Nav1.7) on C nociceptors [15–17]. The sustained activation of TRPA-1 channels has been recognized as a contributory element in developing early diabetic hypersensitivity [14]. This mechanistic interplay underlines the need for therapeutic interventions targeting these molecular pathways to ease neuropathic pain in people with T2DM.

Related to the issue, Andaliman (*Zanthoxylum acanthopodium* DC) has been identified as a promising candidate for further research. Native to the North Sumatra region, Indonesia, the citrus plant has been traditionally utilized for its medicinal properties. Recent studies have reported various pharmacological effects of *Z. acanthopodium* fruit extracts, such as anti-inflammatory, antibacterial, antioxidant, and antidiabetic activities [18,19]. Recent evidence suggests that the fruit’s ethanol extract exhibits anti-inflammatory effects on several proinflammatory mediators, such as TNF-α. These findings indicate that *Z. acanthopodium* fruit extracts could have beneficial therapeutic effects for various inflammation-related disorders, including DN [20]. Because of TNF-α involvement in the progression of DN, insulin’s inhibition of TNF-α or other flavonoid chemicals found in the Andaliman fruit can potentially lessen DN discomfort [21].

In light of these developments, the present study aims to bridge the knowledge gap by investigating the effect of ethanol-extracted Andaliman fruit on TNF-α and TRPA-1 levels within a T2DM-induced mouse model. This research is poised to expand the current understanding of DN pathophysiology and explore the therapeutic potential of Andaliman, thus contributing to the broader quest for effective treatments for DN in the context of T2DM.

## Materials and Methods

### Ethical approval

The research protocols were approved by the Universitas Prima Indonesia’s Institutional Review Board (approval number: 029/KEPK/UNPRI/X/2022).

### Animals

Male albino Wistar rats (*Rattus norvegicus*), aged two months with body weights (BWs) ranging from 200 to 250 mg, were utilized in this research. The mice were kept in a controlled environment with a temperature of 21°C ± 1°C, humidity levels of 55% ± 10%, and a 12 h light/dark cycle. Before treatment, they were acclimated for 2 weeks. The rats were given a 20 gm/day diabetogenic high-fat diet (HFD) for 12 weeks to induce a diabetic state. On the first day of the ninth week, the mice underwent an 18-h fasting period (water was available) and were intraperitoneally injected with 30 mg/kg streptozocin (STZ). Ethanol extract from Andaliman fruit was orally administered at 100, 300, and 500 mg/kg doses from the 13th to 16th week. In the final week, the mice were euthanized by cervical dislocation, and 3 ml of blood was collected from the heart. The supernatant was centrifuged for 15 min, and TNF alpha and TRPA-1 levels were measured.

### High-fat diet

The HFD used in the research was formulated following the methodology outlined by Buettner et al. [22]. Specifically, 100 gm of goat fat and 50 gm of egg yolk were mixed with 1,000 gm of mice feed made from corn rice. The goat fat was melted through heating, while the egg yolk was obtained from boiled eggs. The resulting mixture was combined with 1,000 gm of corn rice and fed 20 gm/day/mouse.

### Andaliman (Z. acanthopodium) 

The fruits were rinsed thoroughly with running water and subsequently drained. Afterward, the wet weight of the fruit was weighed. They were then placed in a drying cupboard until they were completely dried. After that, the dried fruits were blended into a fine simplicia powder. The powder was weighed to determine its dry weight and stored in a sealed plastic container.

### Extraction protocol 

To prepare the extract from Andaliman fruit, 800 gm of Andaliman fruit Simplicia powder was macerated in 75 volumes of 96% ethanol for five consecutive days within a sealed container, periodically stirred, under conditions shielded from light. The mixture was subsequently filtered, and the remaining residue was washed with sufficient solvent, stirred, and filtered once more to produce 100 parts of filtrate. The filtrate was collected and allowed to cool down for 2 days in a dark environment. Subsequently, the ethanol was removed through evaporation at temperatures between 40°C and 50°C using a rotary evaporator and concentrated in a water bath to obtain a dense extract [23].

### Phytochemical screening

A qualitative phytochemical analysis was conducted to identify the primary constituents of the simplicia. The tests included the identification of flavonoids, alkaloids, saponins, tannins, glycosides, and steroids/triterpenoids. Such screenings are crucial in studying natural products as they can reveal the presence of pharmacologically active compounds.

### TNF-α and TRPA-1 analyses

For both TNF-α and TRPA-1 analyses, a wash buffer was produced by diluting 30 ml of concentrated solution with 750 ml of distilled water and stirring to homogeneity. Standard solutions were then pipetted into microtubes, achieving concentrations ranging from 10 to 0.15625 ng/ml using a standard buffer. Approximately 1 h before testing, a biotin antibody solution was prepared at a volume of 0.1 ml/well, accounting for a total volume that includes an additional ±0.1–0.2 ml to ensure accuracy, and diluted at a 1:100 ratio with antibody buffer. Similarly, 30 min before testing, a Horseradish Peroxidase-Streptavidin Conjugate (SABC) solution was prepared with a volume calculated at 0.1 ml/well, including the same volume adjustment as the biotin solution, and diluted at a 1:100 ratio with SABC buffer.

During the Enzyme-Linked Immunosorbent Assay procedure, the plate was washed and 100 μl of standards, samples, and zero control were added to each well, followed by a 90-min incubation at 37°C. This was followed by two washes and the addition of 100 μl of the biotin solution to the wells, which were then incubated for 60 min. The plate underwent three washes before 100 μl of SABC solution was administered to each well, followed by a 30-min incubation period in a light-shielded environment. After five washes, 90 μl of TMB substrate was introduced to each well. This step was followed by a 15–30-min incubation, shielded from light, during which a blue color change correlating with concentration was observed. The enzyme-driven process was halted by introducing 50 μl of a stop solution, which caused the solution to change color to yellow. Subsequently, the absorbance was assessed to ascertain the concentrations.

### Experimental design

This experimental study used a between-subjects design with six groups: (1) Normal group, which was not given any treatment; (2) Negative control or T2DM-only group (HFD for 12 weeks + 30 mg/kg intraperitoneal STZ at week 9 + extract suspension solvent from weeks 13 to 16); (3) Positive control group (T2DM + metformin 4,5 mg/kg from weeks 13 to 16); (4) T2DM + Andaliman extract 100 mg/kg from weeks 13 to 16; (5) T2DM + Andaliman extract 300 mg/kg from weeks 13 to 16; (6) T2DM + Andaliman extract 500 mg/kg from weeks 13 to 16. Blood sugar levels and body weight were assessed at weeks 1, 8, 12, and 16, while TNF-α and TRPA-1 levels were assessed at week 16.

### Sample size

The study’s sample size was determined with Federer’s formula: (*t*-1)(*n*-1) ≥ 15, where *t* represents the number of groups and *n* is the number of samples per group. According to this formula, a minimum of four mice per group was required.

To account for potential dropout, the number was corrected with $n'=\frac{n}{(1-f)}$, where *n*’ is the corrected number and *f* is the anticipated proportion dropout. With an anticipated dropout rate of 20%, the corrected sample size was five animals per group, with 30 mice included.

## Results

### Phytochemical screening results

Table 1 summarizes the phytochemical composition analysis results of Andaliman fruit extract, revealing the presence of flavonoids, tannins, saponins, alkaloids, and glycosides. These findings are consistent with previous reports [18,20]. Interestingly, the tests did not detect steroids, potentially due to the polarity of the solvent. Polar solvents, such as hydroalcoholic extracts, cannot attract non-polar compounds like steroids unless the compounds are attached to polar functional groups, such as flavonoids or glycosides [23].

### Body weight

**Figure 1.**
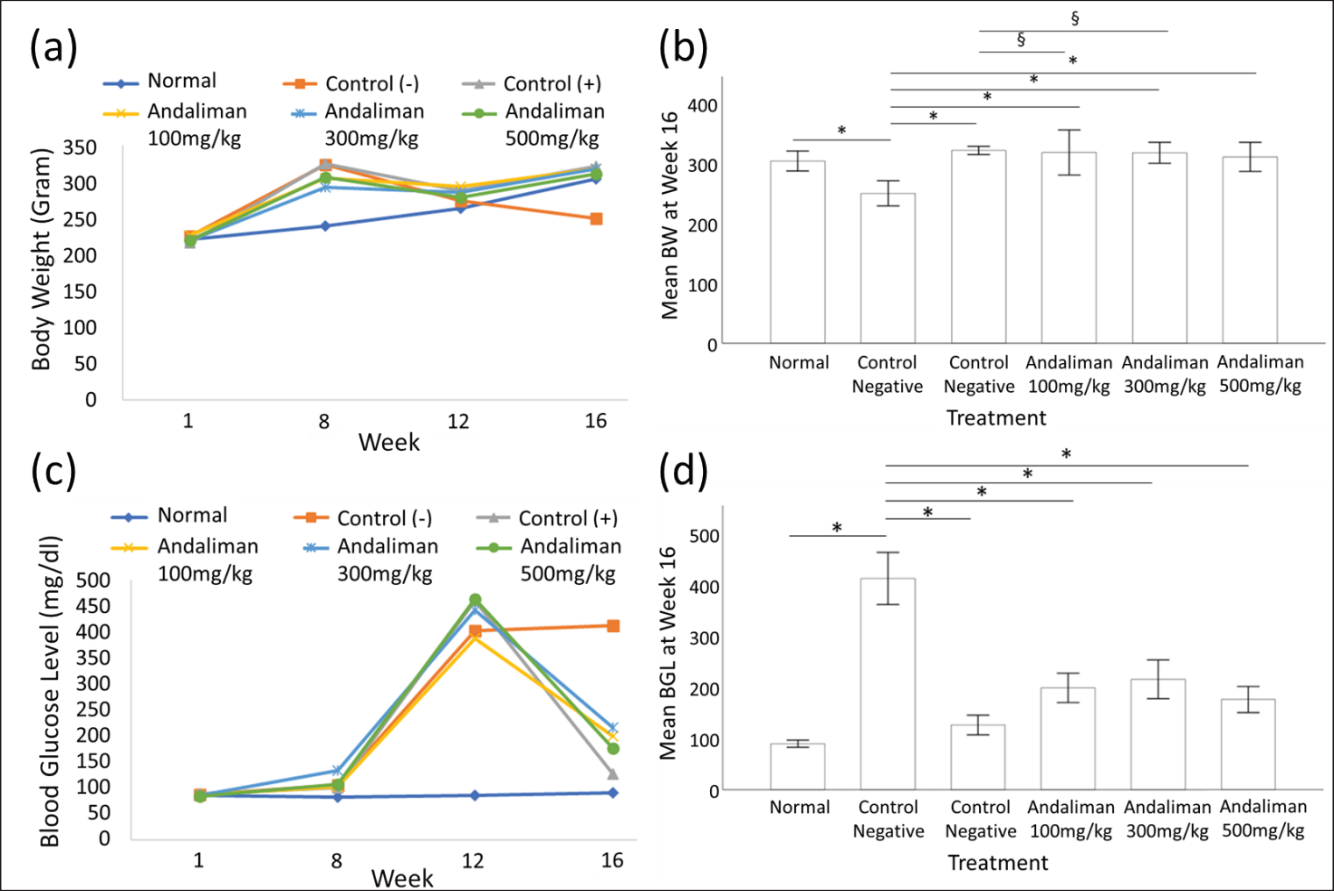
BW and BGLsof T2DM mice (*n *= 26). (a) BW (gm) at weeks 1, 8, 12, and 16; (b) bar chart for week 16’s BGL. *p < 0.05 when compared to negative control; ^§^*p* < 0.05 when compared to positive control; (c) BGL (mg/dl) at weeks 1, 8, 12, and 16; (d) bar chart for week 16’s BW.

Mouse BW was measured at weeks 1 (baseline), 8, 12, and 16, as presented in Table 2. The initial mean BW across groups at baseline showed no statistical difference when analyzed by ANOVA, *p* = 0.850, *F*(5,20) = .39. By week 16, negative control showed a statistically significant reduction in BW compared to the other groups (*p* < 0.05). No statistically significant differences in BW were observed between the positive control and Andaliman groups, *p* > 0.05 (Fig. 1a and b).

### Blood glucose level (BGL)

The mice’s BGL was documented at weeks 1, 8, 12, and 16, detailed in Table 3. At baseline, one-way ANOVA showed no statistically significant difference in BGL across groups (*p* = 0.798, *F*(5,20) = 0.47). By week 16, one-way ANOVA detected a statistically significant difference among the groups, *p* < 0.001, *F*(5,20) = 58.50. Tukey HSD post hoc tests showed that the negative control group’s BGL was significantly higher than the other groups. The other groups (*p* < 0.05). Furthermore, the post hoc test also showed that the 100 and 300 mg/kg Andaliman groups had statistically significantly higher BGL when compared to the positive control (*p* > 0.05). However, no statistically significant difference in BGL was found between the positive control and 100 mg/kg Andaliman, *p *> 0.05 (Fig. 1c and d).

**Table 1.** Results of qualitative phytochemical screening.

Metabolite	Result
Flavonoid	Present
Alkaloid	Present
Tannin	Present
Saponin	Present
Glycoside	Present
Steroid	Absent

**Table 2.** BW (gm) at weeks 1, 8, 12, and 16 (*n* = 26).

Group	Week^a^			
	1	8	12	16
Normal (*n *= 5)	220 ± 13.29	239 ± 15.82	263 ± 13.43	304 ± 18.61*
Negative control (*n* = 4)	225 ± 8.52	323 ± 7.44	273 ± 12.53	250 ± 20.98^§^
Positive control (*n *= 5)	216 ± 9.6	325 ± 20.81	288 ± 17.7	321 ± 7.66*
Andaliman 100 mg/kg (*n* = 4)	227 ± 16.34	305 ± 41.67	293 ± 37.62	318 ± 37.62*
Andaliman 300 mg/kg (*n* = 4)	220 ± 11.84	293 ± 19.97	286 ± 14.48	317 ± 17.48*
Andaliman 500 mg/kg (*n* = 4)	219 ± 18.46	307 ± 6.86	278 ± 26.4	311 ± 24.28*

*Note*.

a Data reported as mean *±* standard deviation.

* *p* < 0.05 when compared to negative control;

§ *p* < 0.05 when compared to positive control.

### TNF-α level

TNF-α levels were assessed at week 16 (Table 4). The TNF-α results were reported using medians because the TNF-α data for the 300 mg/kg Andaliman group violated the assumption of normality of distribution, as indicated by Shapiro-Wilk (*p *> 0.05). The Kruskal-Wallis test conducted to compare the TNF-α levels among the Andaliman groups showed a statistically significant difference in TNF-α levels among the groups (*z* = 22.11, *p* < 0.01). Pairwise comparison with Bonferroni correction showed statistically significant differences between the negative control and the 300 and 500 mg/kg Andaliman groups (*p* = 0.007 and *p* = 0.009, respectively).

**Table 3.** BGL (mg/dl) at weeks 1, 8, 12, and 16 (*N* = 26).

Group	Week^a^			
	1	8	12	16
Normal (*n *= 5)	85 ± 6.8	82 ± 7.12	86 ± 4.69	90 ± 7.85*
Negative control (*n *= 4)	87 ± 5.19	106 ± 4.11	403 ± 73.78	413 ± 50.91^§^
Positive control *(n *= 5)	89 ± 5.45	101 ± 4.85	460 ± 61.63	126 ± 21.54*
Andaliman 100 mg/kg (*n *= 4)	88 ± 2.22	102 ± 10.69	388 ± 37.34	199 ± 28.67*^§^
Andaliman 300 mg/kg (*n* = 4)	86 ± 6.18	134 ± 68.66	442 ± 69.48	216 ± 37.9*^§^
Andaliman 500 mg/kg (*n *= 4)	85 ± 5.68	107 ± 11.47	463 ± 42.39	176 ± 25.54*

*Note*.

a Data reported as mean *±* standard deviation.

* *p* < 0.05 when compared to negative control;

§ *p* < 0.05 when compared to positive control.

### TRPA-1 level

TRPA-1 levels were also assessed at week 16 (Table 5). A one-way ANOVA suggested significant differences in TRPA-1 levels among the groups, *F *(5,20) = 122.84, *p* < 0.01. Given that the data violated the assumption of homogeneity of variance (*p* < 0.05), the Games-Howell was selected for the post hoc tests. The test found that TRPA-1 levels in the negative control were statistically significantly higher than those in the normal control (*p* < 0.001, 95% CI [43.90, 59.61]), the positive control (*p* < 0.001, 95% CI [43.24, 58.95]), 100 mg/kg Andaliman (*p* < 0.001, 95% CI [26.09, 42.64]), 300 mg/kg Andaliman (*p* < 0.001, 95% CI [38.86, 55.41]), and 500 mg/kg Andaliman (*p* < 0.001, 95% CI [42.69, 59.64]). There was a significant dose response, with 300 and 500 mg/kg Andaliman showing greater TRPA-1 reductions than 100 mg/kg (*p *< 0.05). However, no significant differences were found between 300 and 500 mg/kg Andaliman doses (*p* = 0.695, 95%CI [−4.45, 12.11]), indicating a plateau in efficacy beyond 300 mg/kg.

### BGL, BW, TNF-α, and TRPA-1

A Spearman’s rho correlation analysis was utilized to evaluate the correlation between BGLs at week 16, BW at week 16, TNF-α, and TRPA-1 (Table 6). The test was selected because the variables violated the assumption of normality of distribution, as indicated by Shapiro-Wilk tests (*p* < 0.05). The Spearman’s correlation analysis showed statistically significant differences between BGL and TNF-α (*p =* 0.045, *r*_s _(25) = 0.40), BGL and TNF-α (*p < *0.01, *r_s_* (25) = 0.82), and between TNF-α and TRPA-1 (*p* = 0.003, *r_s_* (25) = 0.56). However, no statistically significant correlation was found between BW and TNF-α (*p = *0.094, *r_s_* (25) = −.34) or between BW and TNF-α (*p = *0.68, *r_s_* (25) = −0.36).

**Table 4.** Median TNF-α levels in the experimental groups (*n* = 26).

Group	Median
Normal *(n* = 5)	41.27*
Negative control (*n* = 4)	243.28
Positive control (*n *= 5)	50.27
Andaliman 100 mg/kg (*n *= 4)	167.78
Andaliman 300 mg/kg (*n* = 4)	26.32*
Andaliman 500 mg/kg (*n* = 4)	35.28*

*Note*.

* *p* < 0.05 when compared to negative control.

**Table 5.** TRPA-1 levels in the experimental groups (*n =* 26).

Group	Mean ± S
Normal *(n* = 5)	4.26 ± 1.02*
Negative control (*n* = 4)	56.02 ± 8.80^§^
Positive control (*n *= 5)	4.93 ± 1.10*
Andaliman 100 mg/kg (*n* = 4)	21.66 ± 2.79*^§^
Andaliman 300 mg/kg (*n* = 4)	8.89 ± 1.82*^§ƚ^
Andaliman 500 mg/kg (*n* = 4)	5.06 ± .90*^ƚ^

Note.

* *p* < 0.05 when compared to negative control;

§ *p* < 0.05 when compared to positive control;

ƚ *p* < 0.05 when compared to Andaliman 100 mg/kg.

**Table 6.** Correlation matrix between BGL (week 16), BW (Week 16), TNF-α, and TRPA-1 (*n =* 26).

	BGL	BW	TNF-α	TRPA-1
BGL		−0.27	0.40^*^	0.82^**^
BW			−0.34	−0.36
TNF-α				0.56^**^
TRPA-1				

Notes.

* *p* < 0.01 (2-tailed),

** *p* < 0.05 (2-tailed).

## Discussion

The present study investigated the effects of Andaliman (*Z. acanthopodium*) fruit extract on biological markers relevant to diabetes management, namely BGL, TNF-α, and TRPA-1. During the study, only 26 mice remained alive until the end. Specifically, four mice, including one each from the Negative Control, Andaliman 100 mg/kg, Andaliman 300 mg/kg, and Andaliman 500 mg/kg groups, died at week 9 after STZ induction. This situation is not uncommon since STZ is a genotoxic agent that has been reported to destroy pancreatic beta cells and cause adverse effects in other organs such as the liver and kidneys [24,25]. However, despite these losses, the sample size for each group still adhered to the required minimum sample size.

Phytochemical screening demonstrated the presence of flavonoids, alkaloids, tannins, saponins, and glycosides within the Andaliman fruit extract, aligning with previous literature findings [18–20]. The absence of steroids in the results could likely be due to the polarity of the solvent used in the extraction process, which does not favor the dissolution of non-polar compounds [23]. This finding prompts further consideration of the extraction methods to ensure comprehensive phytochemical characterization.

The study found a statistically significant difference in BGL among the groups at the end of the study, with the negative control group having the highest levels compared to the Andaliman groups. In addition, TNF-α levels were significantly higher in diabetic mice in negative control than those in the Andaliman groups. Meanwhile, the 300 and 500 mg/kg Andaliman groups also had lower levels of TNF-α than the 100 mg/kg Andaliman group, indicating a dose-dependent relationship. Moreover, TNF-α levels in the Andaliman groups were significantly lower compared to the negative control, which is especially relevant given TNF-α’s established role in diabetic complications and inflammation [7,9]. These findings also demonstrate that Andaliman extract could have potential therapeutic effects for individuals with diabetes by reducing BGL and inhibiting the expression of TNF-α and are consistent with prior research on the anti-inflammatory potential of Andaliman extracts [18,21].

TRPA-1, which is implicated in the pathophysiology of DN, was also modulated by Andaliman extract [13,14]. TRPA-1 expression was markedly lower in all Andaliman groups relative to the negative control. The reduction in TRPA-1 was more prominent in the 300 and 500 mg/kg Andaliman groups when compared to the 100 mg/kg group. The positive correlation between BGL and TRPA-1 levels further suggests that Andaliman extract’s regulation of BGL may be associated with TRPA-1 modulation [26]. These findings contribute to the growing body of evidence highlighting the biological link between hyperglycemia and pain perception mediated by TRPA-1 in diabetic conditions.

While the study provides insightful data on the potential benefits of Andaliman extract, several limitations should be acknowledged. The study did not explore the mechanism of action underlying the observed pharmacological effects, which is crucial for understanding how Andaliman compounds interact at the molecular level. Also, mortality following STZ induction, although anticipated, resulted in a reduced sample size that may limit the generalizability of the findings. Moreover, the absence of steroids in the phytochemical analysis may suggest a limitation in the extraction method, potentially overlooking compounds that could contribute to the extract’s therapeutic effects. Finally, the pharmacokinetics of Andaliman compounds, including their absorption, distribution, metabolism, and excretion profiles, were not assessed. Understanding these properties could significantly influence dosage recommendations and safety profiles for future therapeutic use.

## Conclusion

The present study provides evidence for the effects of Andaliman (*Z. acanthopodium*) fruit extract on BGL, BW, TNF-α, and TRPA-1 in the T2DM mouse model. The upregulation of TRPA-1 expression in diabetic rats is highly correlated with the increase of TNF-α. Targeting the TNF-α and TRPA-1 signaling pathways may provide a promising therapeutic strategy for treating T2DM, particularly DN. Further studies investigating the mechanism of action of Andaliman fruit extract on glucose and the regulation of cytokines like TNF-α and TRPA-1 with different doses and durations could provide better insight into managing T2DM, particularly DN.

## List of abbreviations

BGL, Blood glucose level; BW, Body weight; DN, Diabetic neuropathy; HFD, High-fat diet; MGO, Methylglyoxal; SABC, Streptavidin-biotin complex; STZ, Streptozocin; T2DM, Type 2 diabetes mellitus; TMB, Tetramethylbenzidine; TNF-α, Tumor necrosis factor-alpha; TRPA-1; Transient receptor potential Ankyrin 1.
